# Biological impact of different ventilatory strategies during open abdominal surgery in a rat model

**DOI:** 10.1186/2197-425X-3-S1-A568

**Published:** 2015-10-01

**Authors:** L Maia, CDS Samary, MV de Oliveira, CL Santos, R Huhle, M Gama de Abreu, P Pelosi, PL Silva, PRM Rocco

**Affiliations:** Universidade Federal do Rio de Janeiro, Rio de Janeiro, Brazil; Dresden University of Technology, Dresden, Germany; University of Genoa, Genoa, Italy

## Introduction

234 million major surgical procedures are performed worldwide every year. It has been shown that a ventilator strategy with low tidal volume (V_T_), high positive end-expiratory pressure (PEEP) and recruitment maneuvers (RM)s compared to low V_T_ and low PEEP without RM did not protect against postoperative pulmonary complications [1]. So far, no study has evaluated the biological impact of these ventilator strategies in lung tissue during open abdominal surgery. We hypothesized that low V_T_ and low PEEP without RM may result in less biological impact in lung tissue compared to a ventilator strategy with low V_T_, high PEEP and RMs.

## Objective

To evaluate the impact of different ventilator strategies on respiratory system mechanics and biological parameters during open abdominal surgery in rats.

## Methods

28 male Wistar rats (394 ± 60 g) were anesthetized, paralyzed, and mechanically ventilated. After baseline data collection, a laparotomy with bowel manipulation was performed. After this, animals were randomly assigned to one of four groups (n = 7/group): 1) moderate PEEP (3 cmH_2_O), low V_T_ (7 mL/kg), and RM [continuous positive airway pressure (30 cmH_2_O, 30 s) every hour; 2) high PEEP (6 cmH_2_O), low V_T_ (7 mL/kg) and RMs (at the beginning and at the end of the experiment]; 3) low PEEP (1 cmH_2_O), low V_T_ (7 mL/kg) without RMs; 4) low PEEP (1 cmH_2_O), high V_T_ (14 mL/kg) without RMs. All animals were mechanically ventilated for four hours. Respiratory system and lung elastances (E,_RS_ and E,_L_, respectively), peak airway pressure (Ppeak,_RS_), peak transpulmonary pressure (Ppeak,_L_), and blood gas analysis were evaluated every hour. At the end of the experiment, lungs were removed for molecular biology analysis (gene expression of biological markers associated with inflammation (interleukin (IL)-6, damage inflicted pulmonary stretch (amphiregulin) [2], and fibrogenesis (type III procollagen (PCIII))).

## Results

All animals improved oxygenation during the time course of the experiment regardless of ventilator strategy. E,_RS_, E,_L_, Ppeak,_RS_, and Ppeak,_L_ were lower in groups 1 and 2 (submitted to RMs) compared to groups 3 and 4 (no RMs) after 4 hours mechanical ventilation. IL-6 expression increased in all groups independent of the ventilator strategy. Amphiregulin expression was more reduced in group 3 (low PEEP (1 cmH_2_O), low V_T_ (7 mL/kg) without RMs) compared to other groups. PCIII mRNA expression was more increased in group 4 (low PEEP (1 cmH_2_O), high V_T_ (14 mL/kg) without RMs) than other groups.

## Conclusion

Even though groups ventilated to low V_T_, moderate and high PEEP levels, and submitted to RMs improved lung function, they were associated with higher amphiregulin expression. Based on functional and molecular parameters, an intraoperative protective ventilation strategy should include a low V_T_ and low PEEP, without RM.

## Grant Acknowledgment

CNPq, FAPERJ, CAPES, PRONEX
